# Supplementary material for: High-fidelity entanglement between a trapped ion and a telecom photon via quantum frequency conversion
Source: Nat Commun. 2018 May 21;9:1998. doi: 10.1038/s41467-018-04341-2 (PMC5962555; doi:10.1038/s41467-018-04341-2)
Supplement: Supplementary file 1 — Supplementary Information [file 41467_2018_4341_MOESM1_ESM.pdf]

Supplementary information: High-fidelity entanglement between a trapped ion and a telecom photon via quantum frequency conversion, Bock et al.

Supplementary Note 1

Experimental sequence for entanglement generation and atomic state analysis

The experimental sequence, depicted in Supplementary Figure 1, starts with 8 µs of Doppler cooling on the $S_{1/2} \rightleftarrows P_{1/2}$ transition, including repumping of population from the $D_{5/2}$ manifold back to the $S_{1/2}$ ground state, resulting in a mixture between the $|m = +1/2\rangle$ and $|m = -1/2\rangle$ Zeeman sublevels of $S_{1/2}$. As shown in Supplementary Figure 1a, a laser pulse at 393 nm of 3 µs duration excites the ion to $P_{3/2}$, where the polarization and geometry of the pulse are adjusted to drive solely the atomic π transition. Subsequent decay into the metastable $D_{5/2}$ manifold creates a mixture of entangled atom-photon states

$$|\Psi_1\rangle = \sqrt{\frac{2}{3}}\,|R, m = -3/2\rangle + \sqrt{\frac{1}{3}}\,|L, m = 1/2\rangle \tag{1}$$

and

$$|\Psi_2\rangle = \sqrt{\frac{1}{3}}\,|R, m = -1/2\rangle + \sqrt{\frac{2}{3}}\,|L, m = 3/2\rangle \tag{2}$$

where the imbalance in the amplitudes originates from unequal Clebsch-Gordan coefficients for the respective decay channels. Potential decay to $D_{3/2}$ via the emission of a photon at 850 nm is detected as false positive events and thus eliminated in a later step.

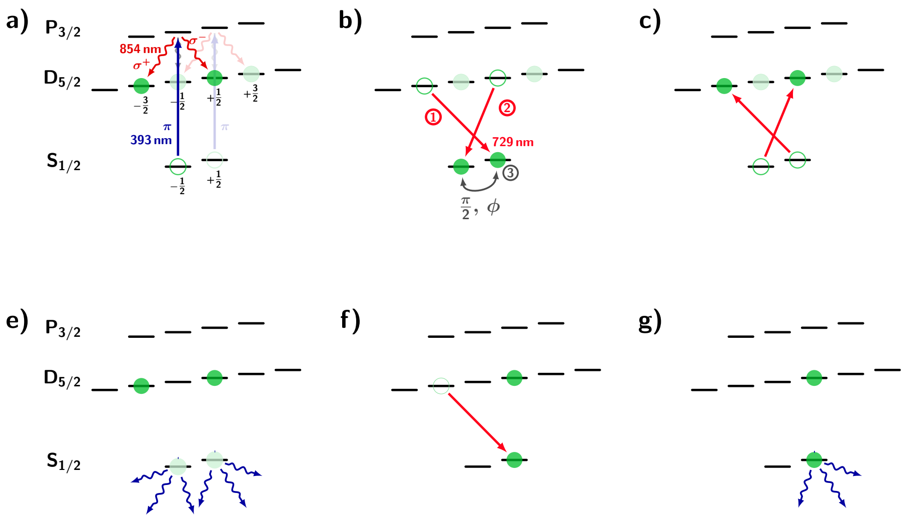

Supplementary Figure 1: Experimental sequence. (a) Starting from a mixture in the ground state, a mixture of two entangled atom-photon states is generated using a π-polarized laser pulse at 393 nm. The emitted 854 nm photon is collected along the quantization axis. (b) Conditioned on the detection of the emitted photon, an optional RF basis-rotation pulse is applied to determine the basis for the atomic state analysis. (c) The population is shelved back to $D_{3/2}$. (d)-(e) Elimination of the unwanted superposition fluorescence-based state discrimination. (f) Population in $|D_{5/2}, m = -3/2\rangle$ is transferred into the $S_{1/2}$ ground state. (g) State read-out by fluorescence detection.

As shown in Supplementary Figure 1b, conditioned on the detection of an 854 nm photon, optional state rotation for the atomic state analysis is carried out by two 729 nm pulses (50 µs each) that coherently transfer the $|m=-3/2\rangle$, $|m=1/2\rangle$ superposition into $S_{1/2}$, followed by a $\pi/2$ radio-frequency (RF) pulse (5 µs), resonant on the $|S_{1/2}, m=-1/2\rangle \rightarrow |S_{1/2}, m=+1/2\rangle$ transition, which translates the superposition phase into population of $|S_{1/2}, m=\pm1/2\rangle$. After shelving the population back to $D_{5/2}$ (Supplementary Figure 1c), the undesired superposition $|m=-1/2\rangle$, $|m=3/2\rangle$ is projected out by transferring the latter to $S_{1/2}$ and eliminating these cases by means of fluorescence detection (200 µs) where "bright" events are omitted (Supplementary Figure 1d/e). In the course of the fluorescence detection, erroneous 850 nm-photon–detection events are eliminated likewise, as population in $D_{3/2}$ leads to fluorescence when the cooling lasers (397 nm and 866 nm) are switched on. State detection of the remaining $D_{5/2}$ populations is conducted by two further 729 nm π pulses coupling $|D_{5/2}, m=-3/2\rangle$ and $|D_{5/2}, m=1/2\rangle$ to $|S_{1/2}, m=1/2\rangle$ with a fluorescence detection after each pulse (Supplementary Figure 1f/g).
The generation sequence takes 11 µs (8 µs for cooling and 3 µs for generation), which yields a maximum achievable sequence rate of about 90.9 kHz. However, due to the atomic state analysis, triggered upon a photon detection event, the rate is reduced to about 58 kHz on average.

Supplementary Note 2
Detailed setup of the quantum frequency converter

A detailed drawing of the converter, which contains all optical elements and the complete setup for the path length stabilization, is shown in Supplementary Figure 2.

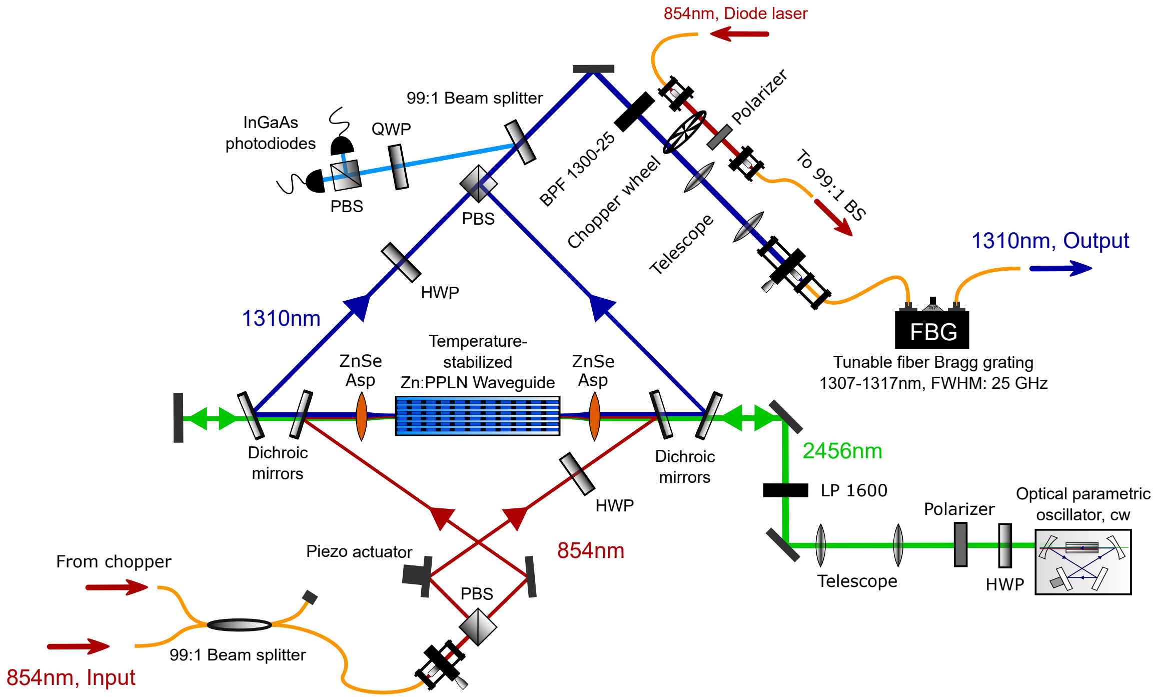

Supplementary Figure 2: Detailed drawing of the polarization-preserving frequency converter HWP: half wave plate, QWP: quarter wave plate, PBS: Polarizing beam splitter, LP: Long-pass filter, BPF: Band-pass filter, ZnSe Asp: Zinc selenide aspheric lens

Supplementary Note 3
External conversion efficiency

We define the external efficiency as the ratio between 1310 nm photons leaving the output fiber of the converter (see blue output arrow in Supplementary Figure 2) and 854nm photons entering the input fiber of the converter (red input arrow in Supplementary Figure 2). Hence it includes the internal conversion efficiency and all transmission and filtering losses between these two fibers, as will be explained in more detail below. We can also interpret the external efficiency as a device efficiency of the QFC when it is applied as a component of a quantum network. Due to the asymmetry of the setup, the individual efficiencies and losses are different in the interferometer arms, which makes a separate consideration of both arms reasonable. All losses and efficiencies were measured individually using the 854 nm alignment laser and the converted signal thereof.
The external conversion efficiency of the H-polarized arm (the arm containing the half wave plates) is composed of the following individual efficiencies:

$$\eta_{\text{ext}_H} = \eta_{\text{WGCoup}} \cdot \eta_{\text{InEff}} \cdot \eta_{\text{DM}} \cdot \eta_{\text{BPF}} \cdot \eta_{\text{FCoup}} \cdot \eta_{\text{FBG}} \cdot \eta_{\text{OpEle}} \cdot \eta_{\text{Asym}} = 26.5(2)\% \tag{3}$$

η_{WGCoup} denotes the coupling efficiency of the input field at 854 nm to the waveguide of 79.7%. This value is limited by the non-optimal overlap between the Gaussian mode of the input light and the astigmatic mode of the waveguide. An improvement might be achieved with cylindrical lenses, which is in our case not possible due to limited space in the 854 nm beam path. The internal conversion efficiency η_{InEff} is defined as the amount of input light which is converted inside the waveguide including losses in the waveguide but excluding losses outside. Not that the internal efficiency is not identical to the signal depletion, which does not take into account losses in the waveguide. We measure an internal efficiency of 96.6% limited by the spatial mode overlap of input, pump and output field. The joint transmission of the dichroic mirrors (each beam has to pass three of them) is $\eta_{\text{DM}} = 81.6\%$. This rather poor transmission is explained by the non-standard angles of incidence, which are outside the specification of the mirror coatings (the dichroic mirrors are specified for 45° angle of incidence). Further transmission losses arise from the spectral filters, namely the band-pass filter with $\eta_{\text{BPF}} = 97.1\%$ and the fiber Bragg grating with $\eta_{\text{FBG}} = 69.5\%$. The coupling to the single-mode fiber $\eta_{\text{FCoup}} = 82\%$ is again limited by a non-perfect mode-matching. All losses in the remaining optical elements (lenses for fiber-coupling, ZnSe-lenses, polarizing beam splitters, 99:1 beam splitters for stabilization, spherical lenses for mode matching) are combined in $\eta_{\text{OpEle}} = 82.5\%$. Due to a higher external efficiency of this arm, it was necessary to decrease the efficiency on purpose with the second HWP to ensure an equal conversion efficiency for both polarization components. This is represented by the asymmetry-correction factor $\eta_{\text{Asym}} = 92.4\%$.

We calculate the external conversion efficiency of the V-polarized arm similarly:

$$\eta_{\text{ext}_V} = \eta_{\text{WGCoup}} \cdot \eta_{\text{InEff}} \cdot \eta_{\text{DM}} \cdot \eta_{\text{BPF}} \cdot \eta_{\text{FCoup}} \cdot \eta_{\text{FBG}} \cdot \eta_{\text{OpEle}} \cdot \eta_{\text{Asym}} = 26.6(2)\% \tag{4}$$

with the waveguide coupling efficiency $\eta_{\text{WGCoup}} = 78.2\%$, the internal conversion efficiency $\eta_{\text{InEff}} = 89.6\%$, the transmission through the dichroic mirrors $\eta_{\text{DM}} = 86.6\%$, the spectral filter transmission $\eta_{\text{BPF}} = 97.1\%$ and $\eta_{\text{FBG}} = 69.3\%$, the fiber-coupling $\eta_{\text{FCoup}} = 77.8\%$ and $\eta_{\text{OpEle}} = 83.8\%$. Apart from small differences in the coupling efficiencies and the losses in all optical elements, a reduced internal efficiency is observed. This is caused by the lack of pump power in the second pass through the waveguide.

Potential improvements to boost the external conversion efficiency are waveguides with a quadratic cross-section yielding a higher overlap with Gaussian modes; a pump source with higher output powers $\geq 2\,\text{W}$ enabling pumping from both sides, which should remove the

remaining asymmetries; dichroic mirrors with coatings designed for the given wavelength combination, polarization and angles of incidence which increase the transmission above 90% and volume Bragg gratings with efficiencies around 95% replacing the fiber Bragg grating. With these improvements, overall efficiencies around 50% are within reach.

Supplementary Note 4
Entanglement generation rate

Measurement without frequency conversion

In this section, the values for the entangled-state generation rate given in the main text are calculated. In the measurement without the frequency conversion, 114,200 events were detected in 4,132 s. Note that only the signal events excluding the APD dark counts are considered for the generation rate. The detection time window is 300 ns corresponding to 65% of the photon wavepacket. Thus we get for the detected entanglement rate after projection

$$\gamma_{854,\mathrm{det}} = \frac{114,200\,\mathrm{events/s}}{4132\,s} = 27.64\,\mathrm{events/s} \tag{5}$$

The generated rate is calculated to

$$\gamma_{854,\mathrm{gen}} = \frac{27.6\,\mathrm{events/s}}{\eta_{\mathrm{Pol,trans}} \cdot \eta_{\mathrm{Pol,ab}} \cdot \eta_{\mathrm{APD}}} = 236\,\mathrm{events/s} \tag{6}$$

with the transmission of the polarizer $\eta_{\mathrm{Pol,trans}} = 78\%$, the quantum efficiency of the APD η_{APD} = 30% and an additional factor $\eta_{\mathrm{Pol,ab}} = 50\%$ as the emitted photons are partially unpolarized, resulting in the absorption of half of the photons on average by the polarizer.
As a check for consistency we calculate the theoretical rate we expect from the sequence repetition rate and the losses/efficiencies between the ion trap and the projection setup. With the repetition rate $\gamma_{\mathrm{rep}} \approx 58\,\mathrm{kHz}$ the generated rate is

$$\gamma_{854,\mathrm{gen,theo}} = \gamma_{\mathrm{rep}} \cdot \eta_{\mathrm{Halo}} \cdot \eta_{\mathrm{Fiber}} \cdot \eta_{\mathrm{Mix}} \cdot \eta_{850} \cdot \eta_{\mathrm{Phot}} \approx 238\,\mathrm{events/s} \tag{7}$$

with the collection efficiency of the HALO $\eta_{\mathrm{Halo}} = 3.6\%$ and the fiber coupling efficiency $\eta_{\mathrm{Fiber}} \approx$ 39%. Furthermore three additional factors have to be taken into account. As explained in Supplementary Note 1, the state preparation creates a statistical mixture of two superpositions in the $D_{5/2}$-manifold, thus half of the photons arise from a decay to the unwanted superposition. The factor $\eta_{\mathrm{Mix}} = 50\%$ takes into account this effect. Moreover, a decay to the $D_{3/2}$-state results in the emission of a photon at 850 nm, which is also collected. With the Einstein coefficients of the transitions at 854 nm and 850 nm ($\mathrm{A}_{854} = 1.35\,\mathrm{MHz}$ and $\mathrm{A}_{850} = 0.152\,\mathrm{MHz}$, respectively) the correction factor is $\eta_{850} = \frac{\mathrm{A}_{854}}{\mathrm{A}_{854}+\mathrm{A}_{850}} = 89.9\%$. Finally, $\eta_{\mathrm{Phot}} = 65\%$ takes into account that only a part of the photon wavepacket is considered. Thus, the theoretically expected rate is in very good agreement with the measured rate.

Measurement with frequency conversion

The detected entanglement generation rate including the frequency converter is computed in an analogue way. Note that we utilized two single-photon detectors for the telecom photon detection instead of a single APD for the unconverted photon detection. With 193,120 events detected in 7,779 s we get

$$\gamma_{1310,\mathrm{det}} = \frac{193,120\,\mathrm{events/s}}{7,779\,s} = 24.8\,\mathrm{events/s} \tag{8}$$

The generated entanglement rate for the converted photons is

$$\gamma_{1310,\text{gen}} = \frac{12.4\,\text{events/s}}{\eta_{\text{Tomo}} \cdot \frac{\eta_{\text{SSPD1}}+\eta_{\text{SSPD2}}}{2}} = 43.5(14)\,\text{events/s} \tag{9}$$

with the transmission of the tomography setup $\eta_{\text{Tomo}} = 86.5\%$ and the quantum efficiencies of the SSPDs $\eta_{\text{SSPD1}} = 70(2)\%$ and $\eta_{\text{SSPD2}} = 62(2)\%$.
Again as a consistency check, we calculate the expected rate with the losses and efficiencies in the telecom setup measured with a laser. For the overall transmission efficiency we get

$$\eta_{1310,\text{trans}} = \eta_{\text{Fiber}} \cdot \eta_{\text{ExCon}} \cdot \eta_{\text{Stab}} = 17.5(1)\% \tag{10}$$

with the transmission of the fiber between the labs (includes the coupling to the fiber and the fiber-fiber coupling to the converter) $\eta_{\text{Fiber}} = 75.8\%$, the external conversion efficiency $\eta_{\text{ExCon}} = 26.5(2)\%$ and the duty cycle of the path-length stabilization $\eta_{\text{Stab}} = 87.5\%$. With a sequence repetition rate of $\gamma_{\text{rep}} \approx 61.7\,\text{kHz}$ (the rate is slightly increased as the atomic state analysis is performed less often caused by a lower photon count rate) the expected generated rate is

$$\gamma_{1310,\text{gen,theo}} = \gamma_{\text{rep}} \cdot \eta_{\text{Halo}} \cdot \eta_{\text{Fiber}} \cdot \eta_{\text{Mix}} \cdot \eta_{850} \cdot \eta_{\text{Phot}} \cdot \eta_{1310,\text{trans}} \approx 44\,\text{events/s} \tag{11}$$

which is again consistent with the measured number. A further interesting quantity, which is important for the following SBR calculation, is the ratio of the probabilities to project and detect a photon at 854 nm and at 1310 nm:

$$\frac{\eta_{1310,tot}}{\eta_{854,tot}} = \frac{\eta_{1310,\text{trans}} \cdot \eta_{\text{Tomo}} \cdot \frac{\eta_{\text{SSPD1}}+\eta_{\text{SSPD2}}}{2}}{\eta_{\text{Pol,trans}} \cdot \eta_{\text{APD}}} = 0.427(15) \tag{12}$$

Compared to $\eta_{1310,\text{trans}}$ this value is clearly higher caused by the higher quantum efficiencies of the SSPDs. Note that for the measurement including the projection to the Bell state all numbers have to be multiplied by a factor 2/3 as losses were introduced to compensate the different CGC.

Supplementary Note 5
Signal-to-background ratio

Without the frequency converter we achieve a signal-to-background ratio defined as the total number of signal events (S_{854} = 114,200 in 4,132 s inside a 300 ns window) divided by the total number of background events (BG_{854} = 3,868) of 29.5. The background in this case is solely determined by the APD dark counts, which is clearly visible if we calculate the expected background yielding

$$\text{BG}_{854,\text{ theo}} = \text{DC}_{\text{APD}} \cdot 300\,\text{ns} \cdot \eta_{\text{Mix}} \cdot \eta_{850} \cdot \gamma_{\text{rep}} \cdot 4,132\,s = 3,803 \tag{13}$$

which is in good agreement with the measured background. DC_{APD} denotes the APD dark-count rate of 117.7 photons/s.

A similar result is obtained for the case including the frequency conversion. The number of signal events (S_{1310} = 193,120 in 7,779 s inside a 300 ns window) and background events (BG_{1310} = 7,953) yields a SBR of 24.3. Here the background has two contributions; a major part are the SSPD dark counts (on average 93.5%) and a minor part the conversion-induced noise of 11.4 photons/s (on average 6.5%):

$$\text{DC}_{1310\_1} = \text{DC}_{\text{SSPD1}} + \frac{\text{DC}_{\text{Conv}}}{2} \cdot \eta_{\text{SSPD1}} = 58.7 + \frac{11.4}{2} \cdot 0.7 = 62.7(2)\,\text{photons/s} \tag{14}$$

$$\mathrm{DC}_{1310\_2} = \mathrm{DC}_{\mathrm{SSPD2}} + \frac{\mathrm{DC}_{\mathrm{Conv}}}{2} \cdot \eta_{\mathrm{SSPD2}} = 56.4 + \frac{11.4}{2} \cdot 0.62 = 59.9(2)\,\mathrm{photons/s} \tag{15}$$

A similar calculation reveals that these dark counts determine the total background:

$$\mathrm{BG}_{1310,\,\mathrm{theo}} = (\mathrm{DC}_{1310\_1} + \mathrm{DC}_{1310\_2}) \cdot 300\,\mathrm{ns} \cdot \eta_{\mathrm{Mix}} \cdot \eta_{850} \cdot \gamma_{\mathrm{rep}} \cdot 7,779\,s = 7,935(25) \tag{16}$$

As a conclusion, inserting the frequency converter lowers the SBR from 29.5 to 24.3. Although we detect only $\approx 43\%$ of the photons at 1310 nm, the SBR is not reduced by this factor as the background is also reduced by a factor 0.52, compensating the losses partially. Nevertheless, further improvements of the SBR are reasonable to distribute entanglement over distances of several hundreds of kilometers. On the one hand, the conversion-induced noise can be reduced by spectral filters with smaller bandwidths. Commercial fiber Bragg gratings offer bandwidths down to 1 GHz without a significant decrease in transmission (Ultra narrow-band filter, Advanced optical solutions GmbH), which already increases the SBR by a factor 25 assuming that the noise is equally distributed around the target wavelength. The fundamental limit is the photon bandwidth of 23 MHz, but in this regime a tradeoff between filter transmission and filter bandwidth has to be accepted. On the detector side, recent progress in the development of NbTiN-based superconducting detectors with milli-Hertz dark count rates [1] are very promising to further increase the SBR about two orders of magnitude compared to our results.

Supplementary Note 6
State reconstruction

The state reconstruction is adapted from [2, 3, 4]. We start with the coincidences between photon-detection events and bright events from the atomic state analysis measured in all 36 combinations of projections to the eigenstates of the Pauli operators $\{\pm\sigma_x, \pm\sigma_y, \pm\sigma_z\}$. Supplementary Figure 3a shows an example for the measurement in the eigenbasis σ_z where the 1310 nm photon is projected to $|L\rangle$ and the ion to $|m = -3/2\rangle$ (red curve) and $|m = 1/2\rangle$ (blue curve), respectively. One measurement in the superposition basis is shown in Supplementary Figure 3b (photon projected to $|V\rangle$). The oscillation is related to the Larmor precession of the phase of the atomic superposition in the $D_{5/2}$-manifold. As the time difference between the start of the excitation pulse at 393 nm and the atomic state analysis is constant for each sequence repetition, the phase of the atomic superposition depends on the photon's detection time, i.e. the time at which the atom-photon entanglement is established.

From the 36 measurements we calculate the normalized joint expectation values $S_{i,j} = \langle\sigma_i \otimes \sigma_j\rangle$ in the Pauli base $\{1, \sigma_x, \sigma_y, \sigma_z\}$. This calculation is straightforward for the eigenbases, where we extract solely the number of coincidences (summed up in the 300 ns window) from the curves shown above. In the case of the projection to the atomic superpositions, the expectation values are determined by the phase and visibility of the Larmor fringes, which we obtain via a fit of the data. With the $S_{i,j}$ we get for the density matrix

$$\rho = \frac{1}{4} \sum_{i,j=0}^{3} S_{i,j} \sigma_i \otimes \sigma_j \tag{17}$$

To ensure that the density matrix is physical, a maximum-likelihood estimation [4] is performed afterwards with the linear reconstructed matrix as initial guess. This final density matrix is utitlized to calculate the fidelities and purities stated in the main text and the next section. The error bars of the latter are derived via a finite difference-quotient method assuming a Poissonian distribution of the detection events.

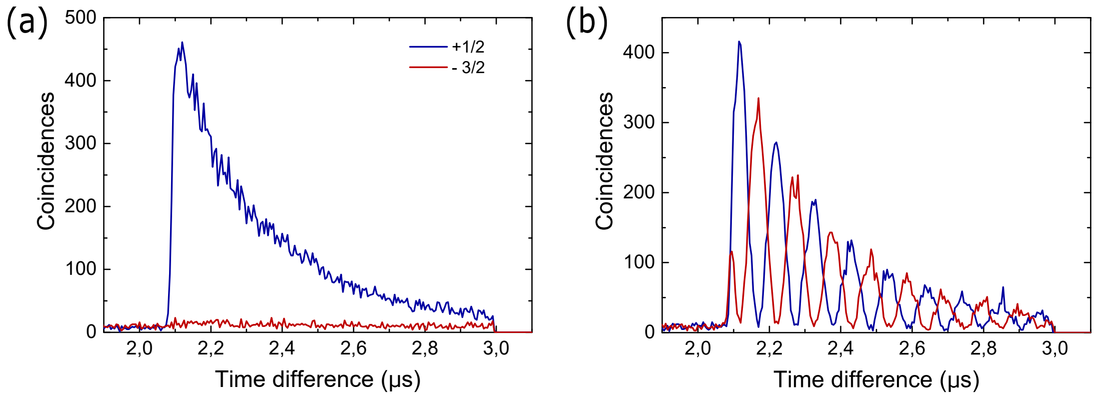

Supplementary Figure 3: Coincidences between 1310 nm photon detection events and bright events from the atomic state analysis. (a) 1310 nm photon and ion are projected onto one of the eigenbases, i.e. the photon is projected to $|L\rangle$ and the ion to σ_z. Thus, no $\pi/2$ radio-frequency (RF) pulse resonant to the $|S_{1/2}, m = -1/2\rangle \rightarrow |S_{1/2}, m = +1/2\rangle$ transition is applied. (b) Projection into one of the superposition bases, i.e. the photon is projected to $|V\rangle$ and the ion to σ_x/σ_y realized by the RF $\pi/2$-pulse.

Supplementary Note 7
Complete list of fidelities and purities

As mentioned in the main text, background subtraction was applied to the raw data to demonstrate the functionality of our method. To perform this background subtraction, we reconstructed the density matrix as described in In Supplementary Note 6 from a modified raw data set. At first, we determine for each basis combination the average number of dark coincidences per time-bin. In Supplementary Figure 3a, for instance, this is done by integrating the number of coincidences outside the photon wavepacket (from 0µs to 2µs) and dividing it by the number of time-bins. This approach is justified in our experiment as we generate triggered single photons within a specified time window; thus, contrary to a photon-pair source, e.g. based on parametric conversion with random emission times, coincidences outside the photon wavepacket originate solely from detector dark-counts. Now, we assume a Poissonian distribution for the dark coincidences (this is feasible as conversion-induced noise as well as detector dark counts occur independently) with the average number of dark coincidences as expectation value. With the distribution of the dark coincidences and the total number of coincidences, we calculate the distribution of the true signal coincidences, yielding expectation value and variance of the latter. From this point, we proceed as described in Supplementary Note 6. This statistical approach is necessary on the one hand to get a proper error estimation and on the other hand to avoid negative values for the number of coincidences in some time-bins.

The following tables summarize again all fidelities and purities with and without subtraction of the background (denoted in the tables as "Total BG subt."' and "W/o BG subt."', respectively). In the measurements with the frequency converter, 93.5% of the background originates from the detectors and 6.5% from conversion-induced noise. Thus it is of interest to quantify solely the converter's influence on the state by subtracting only 93.5% of the background (in this case we multiply the average number of dark coincidences per bin with 0.935). This is denoted in the tables as "Detector DC subt."

Supplementary Table 1: The fidelities denoting the overlap with the expected state

Measurement	Total BG subt.	Detector DC subt.	W/o BG subt.
W/o conversion	98.3 ± 0.3 %	–	95.9 ± 0.3 %
With conversion	97.7 ± 0.2 %	97.3 ± 0.2 %	94.8 ± 0.2 %

Supplementary Table 2: The fidelities denoting the overlap with the Bell state

Measurement	Total BG subt.	Detector DC subt.	W/o BG subt.
W/o conversion	95.5 ± 0.3 %	–	93.3 ± 0.3 %
With conversion	94.8 ± 0.2 %	94.5 ± 0.2 %	92.2 ± 0.2 %
With conv. & state carving	98.2 ± 0.2 %	97.7 ± 0.2 %	93.4 ± 0.2 %

Supplementary Table 3: The purities of all measurements

Measurement	Total BG subt.	Detector DC subt.	W/o BG subt.
W/o conversion	96.7 ± 1.6 %	–	92.1 ± 1.6 %
With conversion	95.8 ± 1.3 %	95.1 ± 1.3 %	90.3 ± 1.2 %
With conv. & state carving	96.7 ± 1.4 %	95.8 ± 1.4 %	87.8 ± 1.3 %

Supplementary References

[1] Schuck, C., Pernice, W. H. P., & Tang, H. X. Waveguide integrated low noise NbTiN nanowire single-photon detectors with milli-Hz dark count rate. *Scientific Rep.* **3,** 1893 (2013).

[2] Kurz, C. *et al.* Experimental protocol for high-fidelity heralded photon-to-atom quantum state transfer. *Nat. Commun.* **5,** 5527 (2014).

[3] Kurz, C., Eich, P., Schug, M., Müller, P. & Eschner, J. Programmable atom-photon quantum interface. *Phys. Rev. A* **93,** 062348 (2016).

[4] James, D. F. V., Kwiat, P. G., Munro, W. J. & White, A. G. Measurement of qubits. *Phys. Rev. A* **64,** 052312 (2001).
